# Identification of Elg1 interaction partners and effects on post-replication chromatin re-formation

**DOI:** 10.1371/journal.pgen.1007783

**Published:** 2018-11-12

**Authors:** Vamsi K. Gali, David Dickerson, Yuki Katou, Katsunori Fujiki, Katsuhiko Shirahige, Tom Owen-Hughes, Takashi Kubota, Anne D. Donaldson

**Affiliations:** 1 Institute of Medical Sciences, University of Aberdeen, Foresterhill, Aberdeen, Scotland, United Kingdom; 2 Centre for Gene Regulation and Expression, University of Dundee, Dundee, Scotland, United Kingdom; 3 Research Center for Epigenetic Disease, Institute of Molecular and Cellular Biosciences, University of Tokyo, Tokyo, Japan; MRC Laboratory of Molecular Biology, UNITED KINGDOM

## Abstract

Elg1, the major subunit of a Replication Factor C-like complex, is critical to ensure genomic stability during DNA replication, and is implicated in controlling chromatin structure. We investigated the consequences of Elg1 loss for the dynamics of chromatin re-formation following DNA replication. Measurement of Okazaki fragment length and the micrococcal nuclease sensitivity of newly replicated DNA revealed a defect in nucleosome organization in the absence of Elg1. Using a proteomic approach to identify Elg1 binding partners, we discovered that Elg1 interacts with Rtt106, a histone chaperone implicated in replication-coupled nucleosome assembly that also regulates transcription. A central role for Elg1 is the unloading of PCNA from chromatin following DNA replication, so we examined the relative importance of Rtt106 and PCNA unloading for chromatin reassembly following DNA replication. We find that the major cause of the chromatin organization defects of an *ELG1* mutant is PCNA retention on DNA following replication, with Rtt106-Elg1 interaction potentially playing a contributory role.

## Author summary

DNA replication is the central process that duplicates the genetic information during cell multiplication. Many cellular factors play important roles in the efficient and accurate duplication of DNA, critical for faithful transmission of genetic information. One such factor is Elg1. Elg1 acts to unload PCNA, the ring-shaped processivity factor that holds DNA polymerases on DNA for replication. In this work, we identify an additional role for Elg1 during replication. We show that lack of Elg1 leads to defects in packaging of DNA into chromatin after DNA replication. In addition, we found that Elg1 interacts with histone chaperones, factors which play key role in chromatin formation. Examining causes of the chromatin re-assembly defect, we show that accumulation of PCNA on DNA is the main cause of defective chromatin formation in the absence of Elg1. By uncovering a new route through which Elg1 ensures chromosomes are perfectly copied, our findings advance understanding of how Elg1 contributes to the stability of the genome through its key roles in DNA replication.

## Introduction

The genetic material in eukaryotes is packaged into chromatin, composed mainly of DNA and nucleosomes. During DNA replication, DNA helicases separate the two parental strands of DNA and nucleosomes are removed from the DNA. Once the nascent DNA strands have been synthesized, the nucleosomal structure must be reassembled to restore the chromatin and permit reinstatement of epigenetic information. Defective chromatin re-assembly leads to improper chromatin formation and loss of epigenetic marks carried on the parental histones, resulting in genomic instability [1].

Various replication-associated factors play a key role in ensuring all the genetic and epigenetic information is efficiently duplicated. A critical component of the replication machinery is PCNA, which serves as the processivity factor for DNA polymerases. Apart from acting as an accessory factor for DNA polymerase, PCNA coordinates replication-associated processes including chromatin re-assembly, cohesion establishment, DNA repair and the damage response [2]. PCNA is loaded onto chromatin during replication by the Replication Factor C (RFC), a pentameric complex consisting of Rfc1-5 [3,4]. During the initiation of each Okazaki fragment, RFC loads PCNA prior to polymerase δ recruitment. On completion of each Okazaki fragment, PCNA must then be unloaded, which requires the Elg1 **R**FC-**L**ike **C**omplex (also called Elg1-RLC; [5,6]. The Elg1-RLC contains the same Rfc2-5 subunits as RFC, but the largest subunit Rfc1 is replaced by Elg1. Timely removal of PCNA is important, and PCNA accumulation in the absence of Elg1 contributes to genomic instability phenotypes such as elongated telomeres, telomeric silencing, chromosomal rearrangements, cohesion defects, and increased sister chromatin recombination [7–11].

Histone chaperones are crucial auxiliary components of the replication machinery [12,13], which ensure the proper coupling of DNA replication with re-assembly into nucleosomes [14]. FACT complex of the budding yeast *S*. *cerevisiae* contains subunits Spt16 and Pob3, and can bind both H2A-H2B and H3-H4. FACT associates with components of the replication machinery including the MCM complex and DNA polymerase δ [15,16] and acts in parental histone recycling and placement on the newly replicated DNA, as well as being implicated in transcription-coupled chromatin control [17,18]. In *S*. *cerevisiae* newly synthesized histone H3-H4 dimers are bound by the histone chaperone Asf1, with new histone H3 preferentially acetylated at H3K56. Asf1 binding and H3K56Ac modification promote the interaction of new H3-H4 with further histone chaperones including CAF-1 and Rtt106 [19], and Asf1 additionally interacts with RFC [20]. CAF-1 is a three subunit complex consisting in yeast of subunits Cac1, Cac2, and Cac3. Two CAF-1 complexes associate to assemble an H3-H4 tetrasome in the initial step of nucleosome re-assembly [21]. CAF-1 promotes nucleosome assembly at replication forks through interaction with PCNA and by binding to DNA directly [22–24]. Rtt106 is also implicated in nucleosome reassembly following DNA replication. Containing two Pleckstrin Homology Domains that mediate its preference for K56-acetylated H3 [21], Rtt106 has been shown to dimerize to mediate assembly of an H3-H4 tetrasome [25,26]. Deletion of *RTT106* when combined with deletion of *CAC1* showed a defect in deposition of H3K56Ac, which is marker of newly deposited histone in yeast [19,27]. Rtt106 is also involved in heterochromatin formation: *rtt106Δ* mutant cells exhibit loss of silencing at mating type loci and telomeres [19,28]. In addition, Rtt106 is proposed to be important for nucleosome assembly during transcription at highly transcribed genes [29] and in regulation of histone gene expression [30,31]. However, it remains unknown how Rtt106 is recruited to required sites of nucleosome assembly.

Because of the links between PCNA and nucleosome assembly, and the effects on chromatin and genome stability caused by *ELG1* deletion [9], we were prompted to investigate whether the PCNA unloading factor Elg1 has a role also in the chromatin re-assembly process. Here we show that Elg1 activity is critical for timely nucleosome organization on nascent DNA. We moreover discovered that Elg1 interacts with histone chaperones, in particular Rtt106 and the FACT complex, with the interaction of Elg1 and Rtt106 not dependent on PCNA. We find however that the most significant cause of defective post-replication nucleosome organization in an *elg1Δ* mutant is delayed unloading of PCNA, with Elg1-Rtt106 interaction potentially playing a contributory role.

## Results

### *In vivo* assays reveal a role for Elg1 in nucleosome assembly

**Fig 1:**
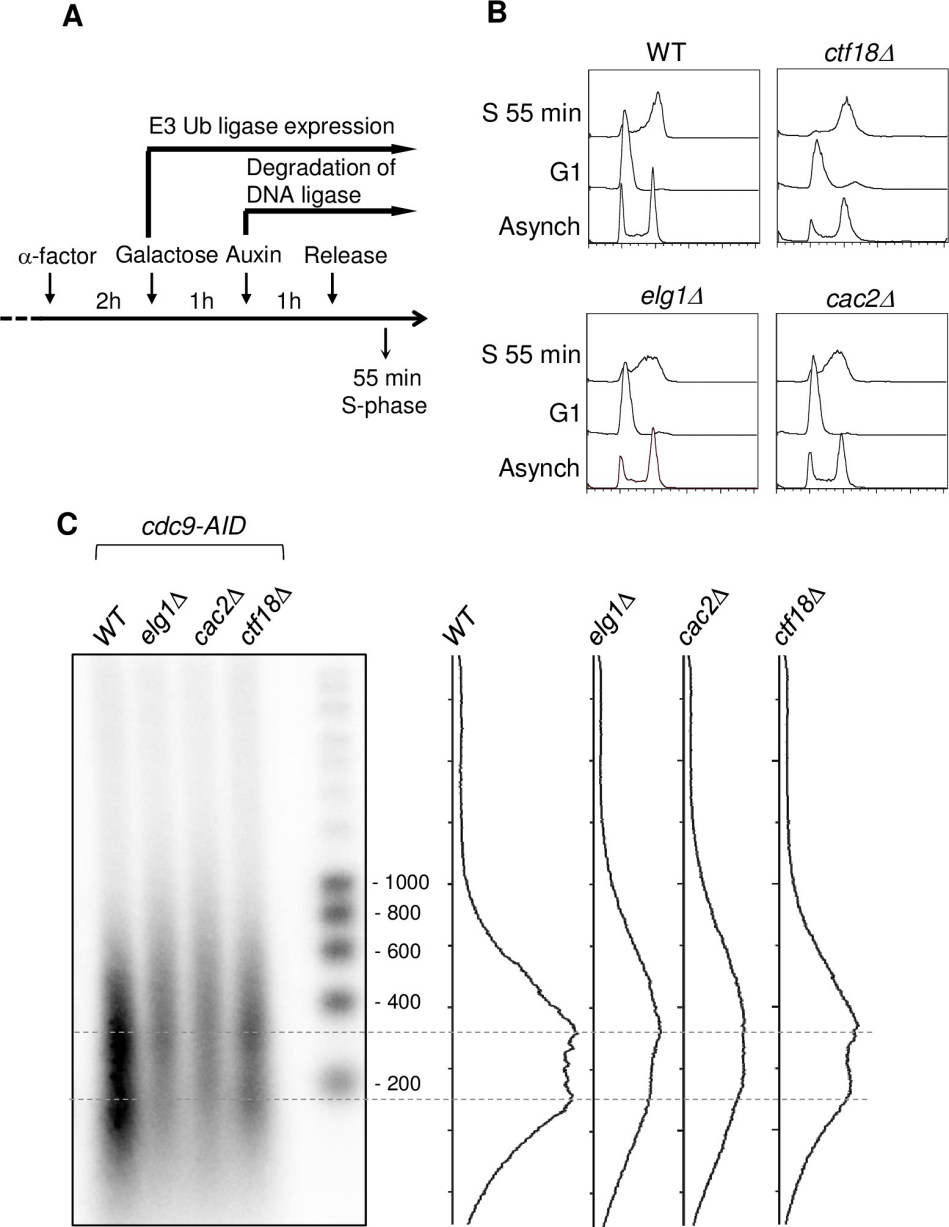
An *elg1Δ* mutant shows extended Okazaki fragments after DNA ligase depletion, suggesting a nucleosome organization defect. A. Outline of the experiment for detection of Okazaki fragment length. B. Flow-cytometry profiles of indicated strains showing progression through S phase. C. Autoradiograph of Okazaki fragments in the strains indicated. Okazaki fragments show extended length in *elg1Δ*, similar to *cac2Δ* and unlike *ctf18Δ*. Dotted lines show Okazaki fragments corresponding to mono- and di-nucleosome sizes. Trace of signal intensity for each lane is shown.

The process of DNA replication and nucleosome re-assembly are tightly coupled. Because it acts at replication forks in PCNA unloading, we examined if Elg1 also affects nucleosome deposition onto newly replicated DNA. Initially, we examined Okazaki fragment length in strains lacking Elg1. Okazaki fragment length can be used as a proxy for nucleosome deposition, since fragment length tends to be determined by the newly deposited nucleosome on the immediately preceding fragment [13,32]. To permit the visualization of Okazaki fragments, we used a strain background with an Auxin-Inducible Degron (AID)-tagged copy of the DNA ligase gene *CDC9*, which accumulates unligated Okazaki fragments during S phase in the presence of auxin. Cells were synchronized in G1 then released into S phase for 55 min, and then Okazaki fragments visualized by 3' end-labelling and gel electrophoresis as described [6,13] (Fig 1A & 1B). In normal cells, Okazaki fragment lengths tend to cluster around 180 bases and 360 bases corresponding to mono- and di-nucleosomal sizes. As previously described, Okazaki fragments are somewhat extended in the mutant *cac2Δ* which lacks the CAF-1 chromatin assembly factor (Fig 1C) [13,32]. This lengthening is believed to reflect aberrant and delayed nucleosome repositioning, which causes continued nick translation and Okazaki fragment lengthening by DNA polymerase δ, since it does not encounter a nucleosome on the previously synthesized DNA that would stimulate its disengagement. In an *elg1Δ* mutant, we found that Okazaki fragment lengths also differed from wild-type, showing a generally broader distribution with a higher proportion of fragments extended in length when compared to wild-type (Fig 1C & S1 Fig). This Okazaki fragment lengthening suggests that the *elg1Δ* mutation may cause a nucleosome assembly defect. The lengthened Okazaki fragment phenotype was not shared by a *ctf18Δ* mutant, which lacks the Ctf18-RLC complex that is involved in establishment of cohesion [33,34]. The effect of Elg1 in limiting Okazaki fragment length therefore appears specific to Elg1-RLC. Since Cdc9 depletion is intrinsic to the Okazaki fragment detection assay, we cannot exclude the possibility that lack of Cdc9 contributes to this Okazaki fragment lengthening effect in the *elg1Δ* mutant.

**Fig 2:**
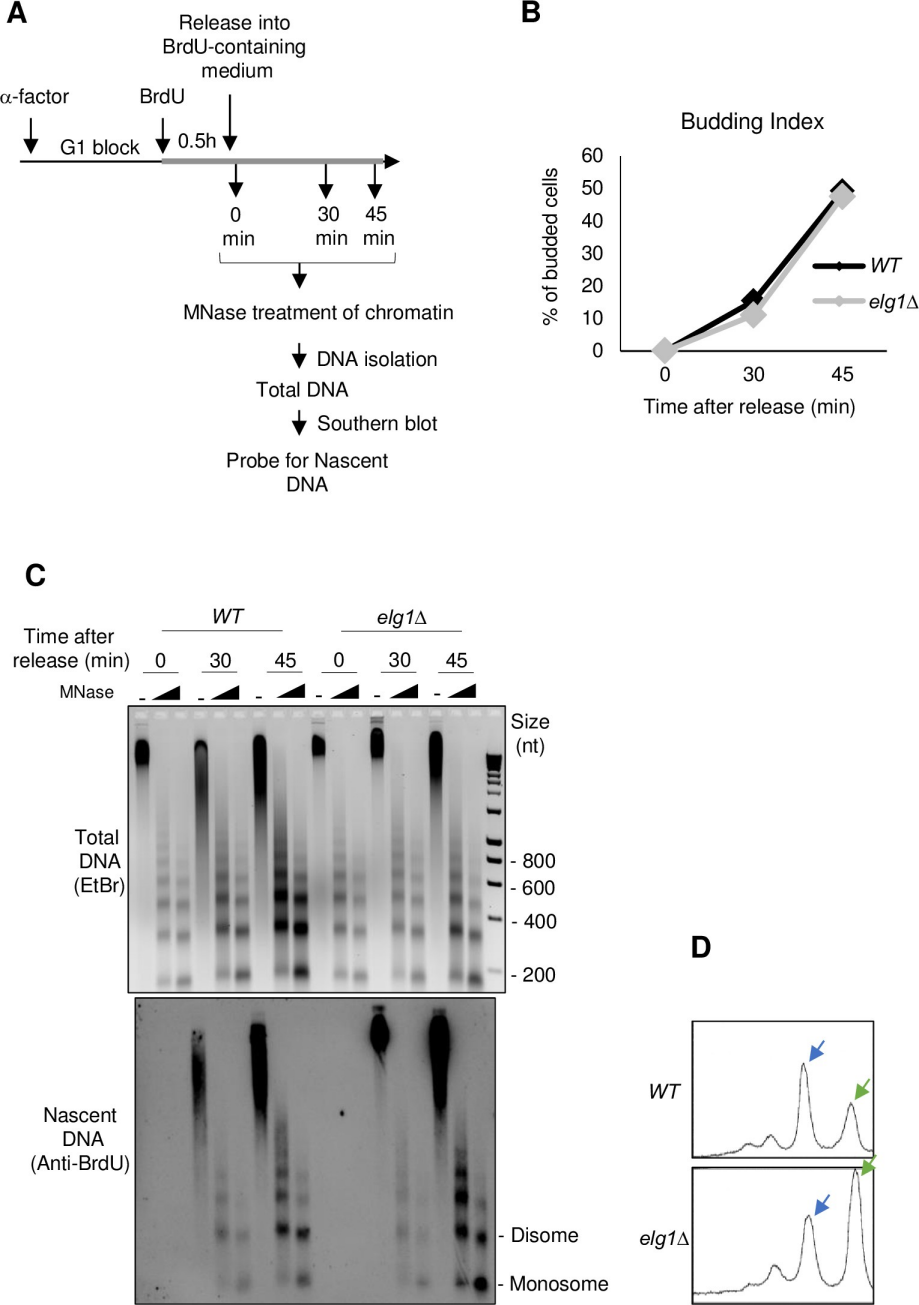
Sensitivity of nascent chromatin in *elg1Δ* to micrococcal nuclease digestion reveals defective nucleosome assembly. A. Outline of experiment. Thick grey line indicates the presence of BrdU in the culture medium. B. Budding index (% of budded cells) in WT and *elg1Δ* indicating synchronous progression through S phase. C. Micrococcal nuclease digestion of chromatin from *WT* and *elg1Δ* strains at indicated times after release into S phase. Upper panel: total DNA on agarose gel detected by Ethidium bromide staining. Lower panel: nascent DNA on membrane probed with anti-BrdU antibody. Micrococcal nuclease (MNase) concentrations: 200 and 600 gel units. D. Signal traces of 45 min, 600 gel units MNase concentration lanes from *WT* and *elg1Δ*. Blue and green arrows indicate di- and mono-nucleosomal peaks.

To examine chromatin re-assembly in *elg1Δ* using a different approach, we next tested the sensitivity of chromatin to digestion by Micrococcal Nuclease (MNase), since defective chromatin re-assembly can result in increased accessibility to digestion by this nuclease. There was no evident abnormality in MNase sensitivity of bulk chromatin in an *elg1Δ* mutant. However, defects in replication-coupled chromatin re-assembly tend to be transient and quickly restored following replication by redundantly acting histone chaperones and/or replication-independent histone turnover [35]. To test nucleosome deposition onto newly replicated DNA, we used cultures synchronized by release from α factor into S phase and examined the MNase sensitivity of nascent DNA labelled with the thymidine analog 5-Bromo 2-deoxyuridine (BrdU) (Fig 2A & 2B). These experiments used strains genetically modified to incorporate BrdU. After Southern blot transfer of MNase-digested DNA to membrane, nascent DNA was specifically visualized by probing the DNA on the membrane with anti-BrdU antibody. Validating the assay, nascent DNA in a *cac1Δ* mutant (S2C Fig) was more sensitive than wild-type to MNase digestion, due to delayed chromatin re-assembly [35]. We found that nascent DNA in the *elg1Δ* mutant (Fig 2C) was also more sensitive to MNase than wild-type, as evidenced by an increased proportion of mononucleosomal compared to disomal digested fragments (Fig 2C lower panel, compare proportion of disome and monosome bands and signal traces of 45 min samples of nascent DNA in Fig 2D). This increased sensitivity to MNase digestion in *elg1Δ* was reproducible, as illustrated by the additional gels shown in S2A & S2B Fig. The magnitude of the effect did vary between experiments: the proportion of mono-nucleosomal to total nascent DNA was increased 1.7-fold in *elg1Δ* relative to wild-type in Fig 2C, 1.2-fold in S2A Fig, and 2.6-fold in S2B Fig. Such variation is to be expected given the semi-quantitative nature of such experiments, but overall the elevated accessibility of nascent DNA to MNase digestion is indicative of defective or delayed nucleosome assembly. The differences in sensitivity to MNase are not caused by different rates of progression through S phase of *WT* and *elg1Δ* cells (S3 Fig). To summarize, our observation of extended Okazaki fragments and increased sensitivity to micrococcal nuclease in the *elg1Δ* mutant suggest a role for Elg1 in replication-coupled nucleosome re-organization.

### Genome-wide analysis reveals delayed nucleosome organization during S phase in *elg1Δ*

**Fig 3:**
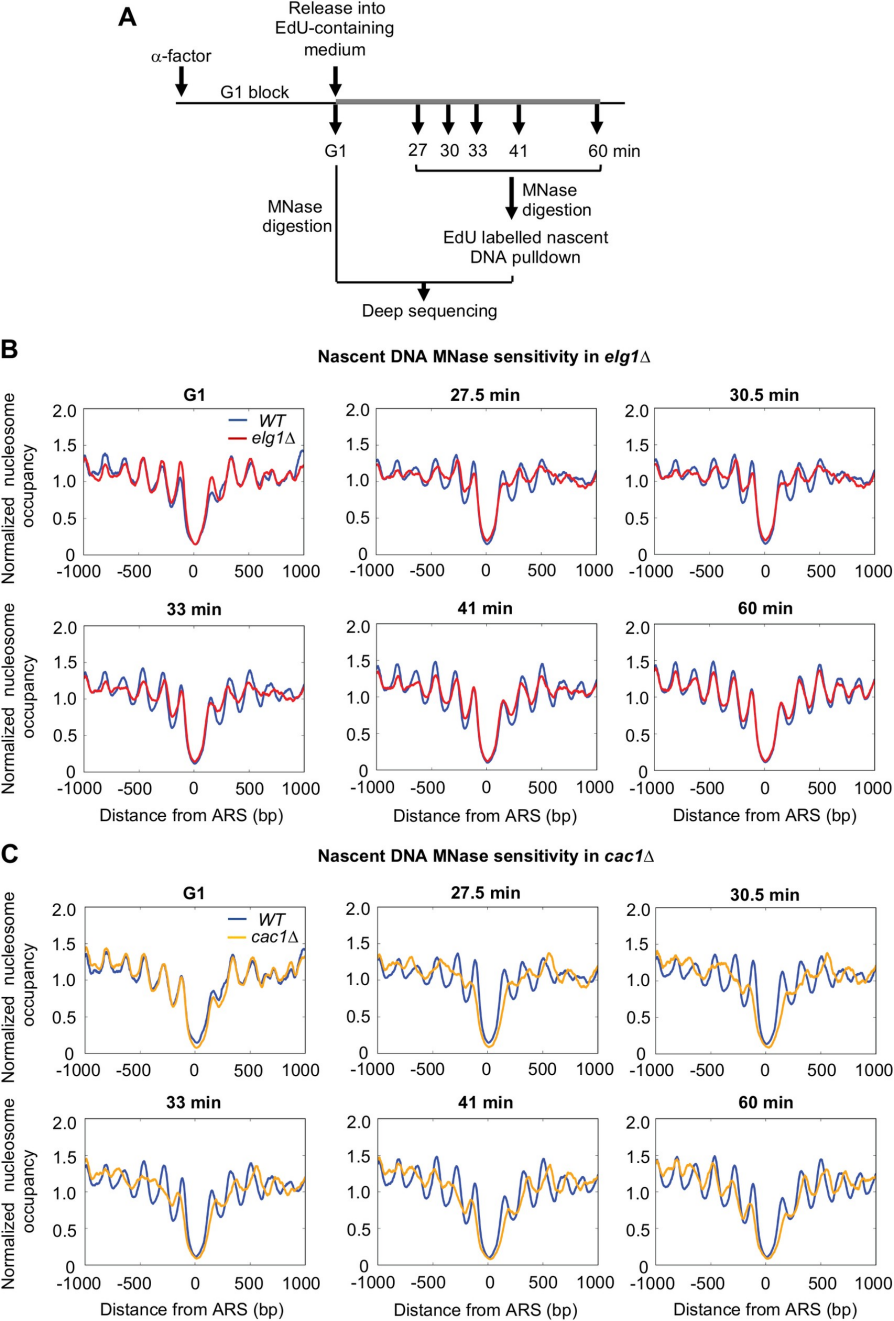
Genome-wide MNase-seq analysis of EdU labelled nascent DNA shows defective nucleosome organization in *elg1Δ*. A. Outline of the MNase-seq experiment. Thick grey line indicates the presence of EdU in culture medium. B & C. Plots showing protection from MNase of EdU-labelled nascent DNA, aligned to origins of replication (ARS sites) in *elg1Δ* (**B**) and *cac1*Δ (**C**) compared to *WT*. Plots in (B) are mean of two biological replicates shown individually in S5 Fig. G1 samples show MNase-digested total DNA. 27.5–60 min samples show MNase-digested nascent DNA recovered by EdU pull down.

The results presented above prompted us to investigate effects of the *elg1Δ* mutation on nucleosome assembly genome-wide. We used thymidine analog 5-ethynyl-2’-deoxy-uridine (EdU) to label newly replicated DNA in G1-arrested cells released into S phase. Following MNase digestion, EdU-labelled nascent DNA was isolated by affinity purification (Fig 3A). After deep sequencing [35], nucleosomal reads were then aligned with respect to origins of replication (Fig 3B & 3C) or transcription start sites (TSS) of all genes (S4 Fig). While no difference in the organization of nucleosomes either upstream or downstream of origins was observed in G1 control samples, a clear defect in organization of nucleosomes is observed in *elg1Δ* (Fig 3B) at early time points after release (27 min, 30 min, 33 min) when compared to WT. As cells reach the end of S phase (60 min) the nucleosomal pattern in the *elg1Δ* mutant becomes more organized and similar to WT, consistent with recovery of normal nucleosome distribution as previously described [35]. Defective nucleosome organization in *elg1Δ* mutant is somewhat similar to that seen in a *cac1Δ* mutant (Fig 3C & S4B Fig) although the *cac1Δ* mutant shows an increased spacing of nucleosomes on nascent DNA that is not obviously shared by *elg1Δ*.

### Elg1 interacts with Rtt106 and other histone chaperones

**Fig 4:**
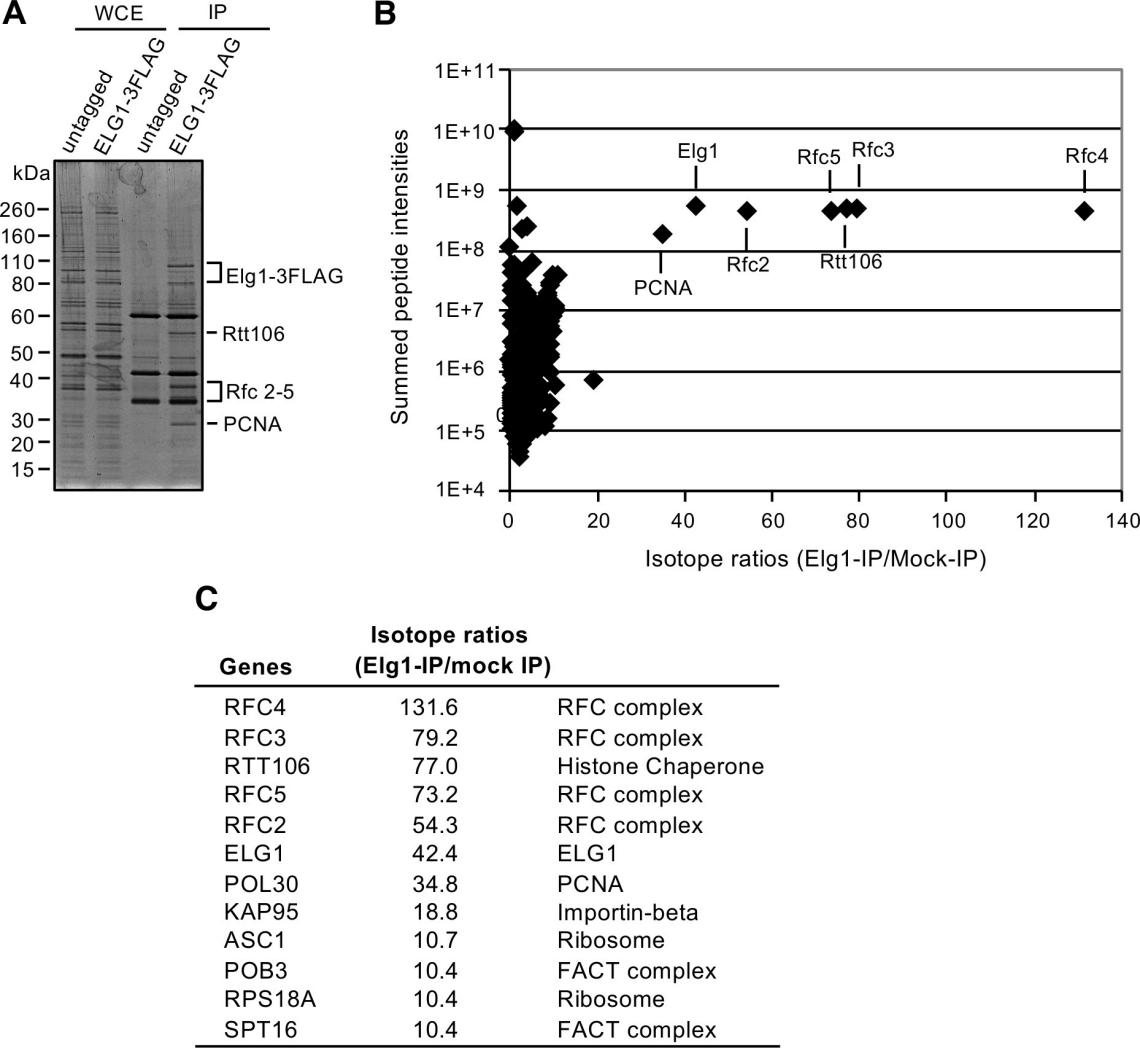
Rtt106 is identified as Elg1-binding protein by SILAC-IP. A. Untagged or Elg1-3FLAG tagged strains were differentially labelled with light or heavy lysine and arginine respectively. Following immunoprecipitation with anti-FLAG antibody, IP samples were analysed by SDS-PAGE followed by SYPRO Ruby staining. B & C. Isotope ratios (Elg1-IP/mock IP) and peptide intensities of the proteins identified by SILAC-IP. Rtt106 is enriched at levels similar to those for the RFC2-5 subunits of Elg1 complex.

To identify interaction partners of Elg1 potentially connected to nucleosome assembly, we used SILAC-based mass spectrometry to identify co-precipitating proteins. Strains expressing untagged or FLAG-tagged versions of Elg1 were differentially labelled with isotopically light or heavy lysine and arginine, and immunoprecipitated proteins (Fig 4A) were analyzed by mass spectrometry. As expected, the Elg1-FLAG samples showed strong enrichment of Elg1 and Rfc2-5 (the other Elg1-RLC subunits) and also of PCNA. Strikingly, the histone chaperone Rtt106 was also enriched at levels similar to the Rfc2-5 subunits (Fig 4B & 4C). Also enriched were Spt16 and Pob3, two subunits of the FACT complex. Both Rtt106 and FACT complex are implicated in replication-coupled nucleosome assembly: while FACT appears to mediate recycling of parental histones, Rtt106 is involved in depositing newly synthesized histones [18,19,36]. The interactions suggest that these histone chaperones, particularly Rtt106, could potentially mediate the nucleosome assembly role of Elg1.

**Fig 5:**
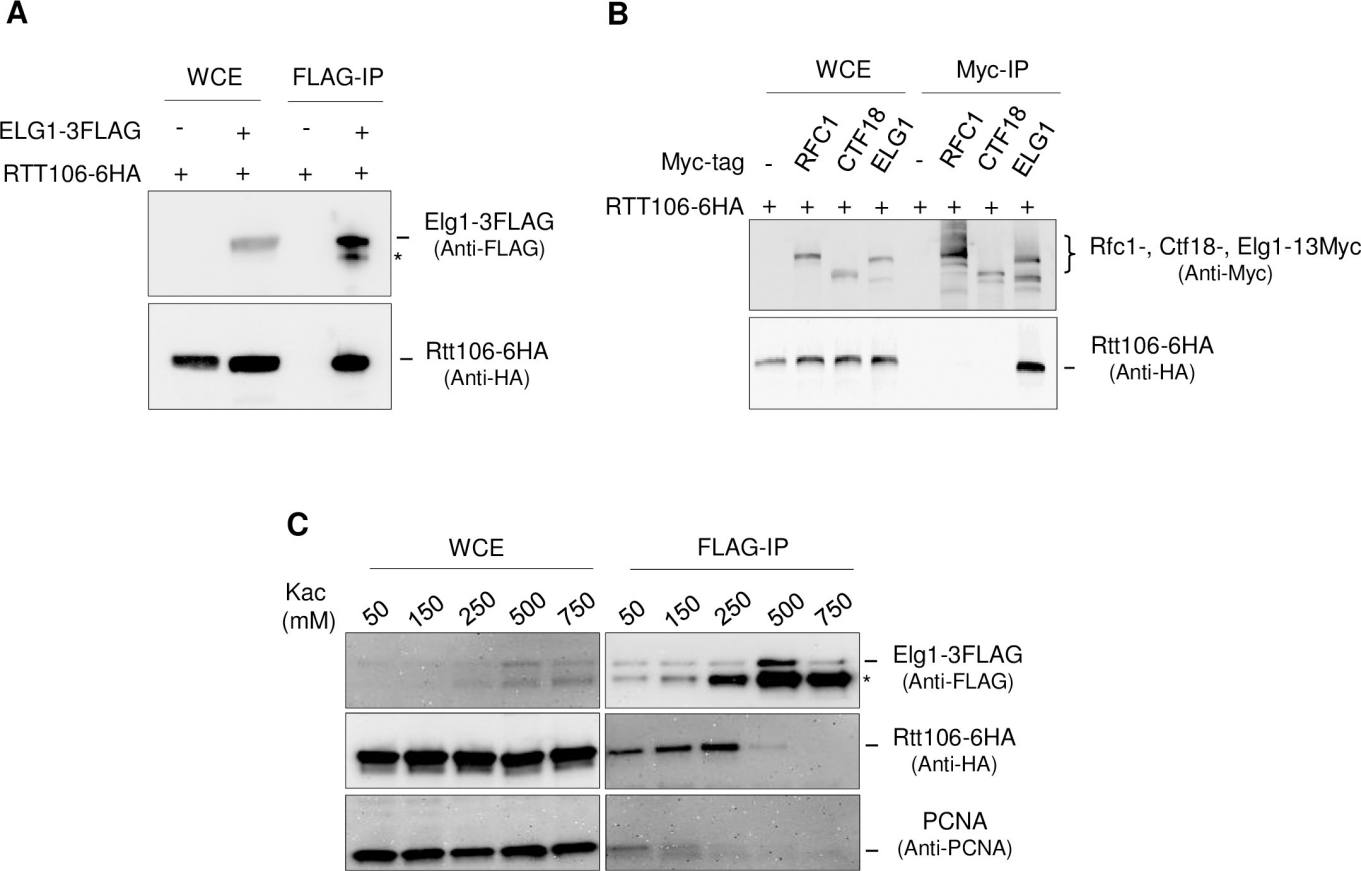
Rtt106 interacts with Elg1 but not other RFC like complexes. A. Confirmation of the interaction between Elg1 and Rtt106 by FLAG-IP and Western blot analysis. Asterisk denotes a degradation product. B. Co-immunoprecipitation experiments showing Rtt106 interacts with Elg1 but not the major subunits of other RFC-like complexes, RFC1 and CTF18. C. Co-immunoprecipitation under different salt concentrations (potassium acetate as indicated) shows interaction of Elg1 with Rtt106 is not mediated by PCNA (also, see S7 Fig). Asterisk denotes degradation product.

We carried out further co-immunoprecipitation experiments with Rtt106 to confirm and investigate the Elg1-Rtt106 interaction. Immunoprecipitation of Elg1-FLAG pulled down HA-tagged Rtt106 (Fig 5A). Pulldown of Elg1 truncation mutants showed that both the Elg1 N-terminal and C-terminal regions are important for the interaction with Rtt106 (S6 Fig). These regions are unique to Elg1, having only very limited sequence similarity with Rfc1 or Ctf18. Consistently, neither Rfc1 nor Ctf18 showed interaction with Rtt106 in co-immunoprecipitation experiments (Fig 5B), suggesting interaction with Rtt106 is a property specific for Elg1 amongst the major subunits of RFC and its related complexes.

Immunoprecipitation of Elg1-FLAG pulled down not only Rtt106 but also PCNA, reflecting the function of Elg1-RLC as the major PCNA unloader. Co-immunoprecipitation experiments in the presence of increasing salt concentrations showed that interaction with PCNA was lost at a concentration where Rtt106-Elg1 interaction was retained (Fig 5C, 250mM potassium acetate & S7 Fig), indicating that the Elg1-Rtt106 interaction is not mediated through PCNA. Note that a band appearing in Western analysis slightly below full-length Elg1 (Fig 5A, 5B & 5C) appears to represent a degradation product whose appearance is stimulated by increased salt concentration. To summarize, our results indicate that robust interaction occurs between Elg1 and Rtt106, specific to Elg1 amongst the RFC-related complexes.

Since Elg1 is important for nucleosome deposition and interacts with Rtt106, we reasoned that, during DNA replication on the lagging strand, Elg1 might concomitantly recruit Rtt106 as it unloads PCNA, thereby coupling PCNA unloading and chromatin re-assembly. Alternatively, Rtt106 might participate in the PCNA unloading function of Elg1. Examining the accumulation of PCNA on chromatin in the absence of Rtt106 (S8 Fig) did not show clear evidence for a role for Rtt106 in PCNA unloading. We therefore followed up the possibility that Elg1 interaction is important to recruit Rtt106 for chromatin re-assembly, by investigating whether recruitment of Rtt106 to replicating regions is dependent on Elg1. We carried out ChIP-seq analysis of HA-tagged Rtt106 on cells released into hydroxyurea from a G1 arrest. However contrary to our expectation, we did not consistently observe association of Rtt106 with newly replicated regions at early origins (e.g. ARS306, ARS510, ARS310, S9B Fig). Nor did we observe convincing Rtt106 recruitment to replicating chromatin in a similar experiment carried out under unperturbed conditions (i.e. in WT cells with no HU treatment). Our ChIP experiments did effectively identify Rtt106 binding as we did observe Rtt106 localization at the promoter *HTA1-HTB1* promoter (S9A Fig), as previously described [37]. Rtt106 recruitment to the *HTA1-HTB1* promoter was not affected in the absence of Elg1 (S9A Fig). We did notice Rtt106 association with the promoters of some genes encoding putative drug exporters, that in some cases appeared Elg1-dependent. This promoter association does not appear replication-linked, since it was observed at some late-replicating regions that forks will not reach under the HU block conditions of the experiment. The importance of Rtt106 promoter binding will be described elsewhere.

### Defective nucleosome organization in the absence of Elg1 is caused mainly by PCNA retention on DNA

**Fig 6:**
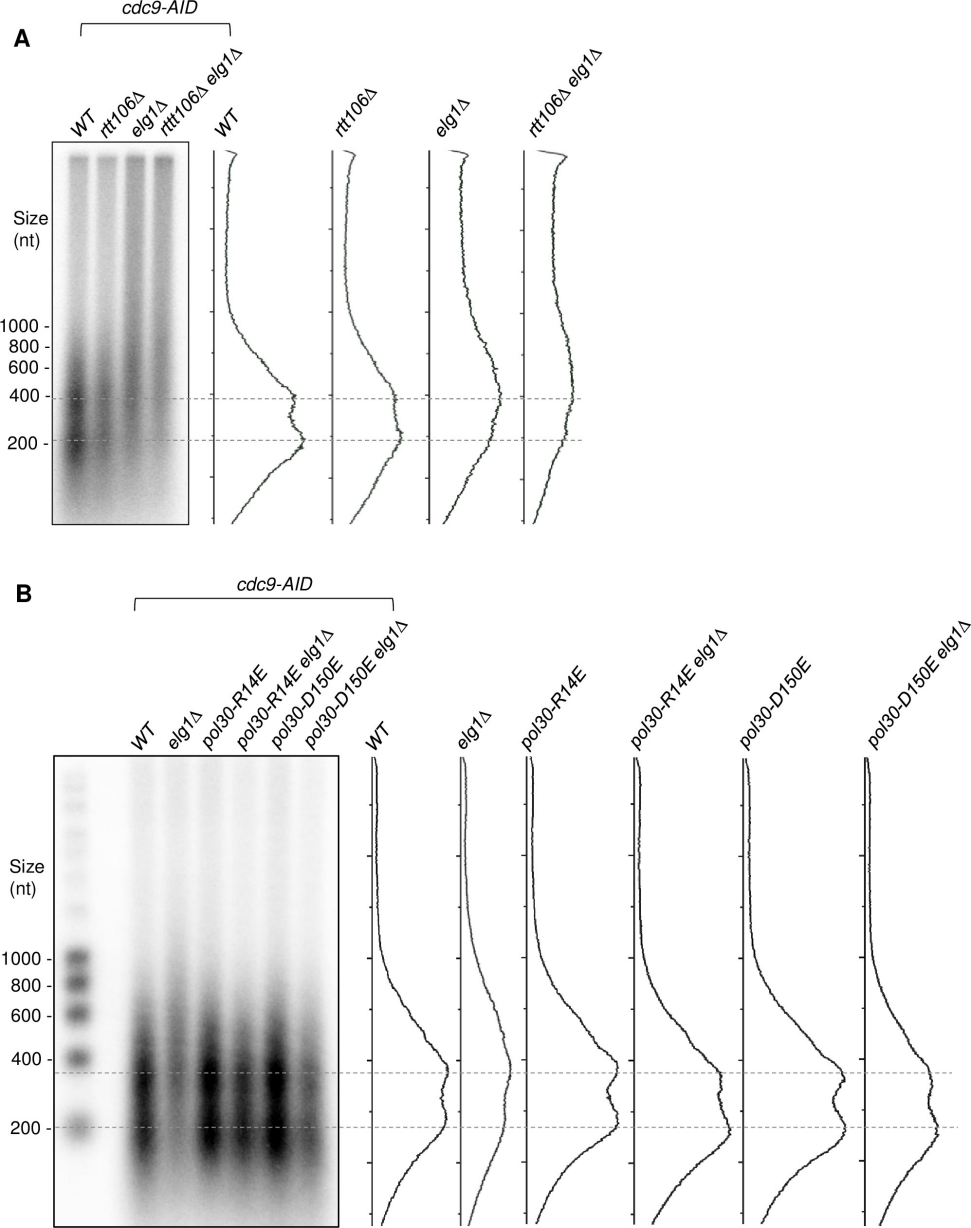
Extended Okazaki fragments in the *elg1Δ* mutant are rescued by disassembly-prone mutants of PCNA. A. Okazaki fragments are extended less in *rtt106Δ* mutant than in *elg1Δ*, suggesting that Elg1 affects Okazaki fragment length independent of Rtt106. B. Disassembly-prone mutant of PCNA (*pol30-R14E* or *pol30-D150E*) rescue the Okazaki fragment length extension observed in *elg1Δ*. Dotted lines show Okazaki fragments corresponding to mono- and di-nucleosome sizes. Trace of signal intensity for each lane is shown.

Given the effect of Elg1 on chromatin re-assembly and its interaction with Rtt106, we tested whether the two proteins act in chromatin re-assembly in the same pathway. Specifically, we examined whether the *elg1Δ* and *rtt106Δ* mutations have similar effects on the length of Okazaki fragments. We found that *rtt106Δ* causes only mild lengthening of Okazaki fragments, the degree of lengthening much less than observed for *elg1Δ*. Moreover, the effect of *elg1Δ rtt106Δ* double mutation on Okazaki fragments appeared to be additive rather than epistatic when compared to the single mutations (Fig 6A). These effects suggest that Elg1 acts in a distinct pathway from Rtt106. Hence, we considered other mechanisms through which *elg1Δ* might affect chromatin re-assembly. The absence of Elg1 results in prolonged accumulation of PCNA on chromatin [11], which could potentially interfere with nucleosome deposition causing defective chromatin re-organization. To investigate this possibility, we made use of trimer instability mutations in PCNA. These mutations cause the PCNA ring to be disassembly-prone, falling off DNA spontaneously even in the absence of Elg1 and thereby suppressing the PCNA accumulation phenotype of the *elg1Δ* mutant [11]. Okazaki fragment length assays were performed in double mutants where *elg1Δ* was combined with two different trimer instability PCNA mutants, *pol30-R14E* (Paul Solomon Devakumar et al. *in revision*) and *pol30-D150E* [11]. We observed that in these double mutants, Okazaki fragments were restored to normal length, when compared to the elongated Okazaki fragments of the *elg1Δ* single mutant (Fig 6B & S10 Fig). Based on this observation, we propose that when normal PCNA unloading fails due to absence of Elg1, aberrant PCNA accumulation on the newly replicated DNA leads to defective nucleosome deposition.

## Discussion

In this investigation, we show that Elg1 contributes to proper nucleosome assembly across the genome after DNA replication, as evidenced by Okazaki fragment lengthening (Fig 1) and elevated sensitivity of nascent DNA to micrococcal nuclease digestion (Figs 2 & 3) in an *elg1Δ* mutant. Okazaki fragment length has previously been examined in several studies as a proxy for nucleosome deposition [32]. This assay could raise the concern that the DNA ligase-deficient background required to visualize Okazaki fragments might itself impact on fragment length or nucleosome re-assembly, but a different study [38] obtained consistent results, also finding that nucleosome position determines *S*. *cerevisiae* Okazaki fragment positioning, using a completely different approach that analyzed mutations inserted by an error-prone polymerase α prone to ribonucleotide insertion. Moreover, in assays that measure the micrococcal nuclease sensitivity of nascent DNA (in cells where DNA ligase activity is intact) we confirmed that nucleosome deposition is affected by the *elg1Δ* mutation. Therefore, the Okazaki fragment lengthening phenotype indeed reflects a nascent strand chromatin re-assembly defect.

To understand interactions that may contribute to the chromatin re-assembly effect of Elg1, we examined the proteins that co-precipitate with Elg1 in pull-down experiments, and identified novel interactions of Elg1 with histone chaperones, in particular Rtt106 and the FACT complex. Interestingly, Rtt106 appears to bind the Elg1-RLC in almost stoichiometric amounts, in an interaction that does not depend on PCNA. Rtt106 does not interact with either Rfc1 or Ctf18. Consistently, we found that both the N-terminal and C-terminal regions that are unique to Elg1 are needed for Rtt106 interaction (S6 Fig).

To examine the extent to which Rtt106-Elg1 interaction versus the Elg1 PCNA unloading function are important for chromatin re-assembly, we made use of disassembly-prone mutants of PCNA which do not accumulate on chromatin even in the absence of Elg1. Using these mutations to relieve PCNA accumulation on chromatin in an *elg1Δ* background restored Okazaki fragments to normal length, indicating that prompt and effective PCNA unloading is absolutely essential for normal nucleosome deposition in the wake of replication forks.

**Fig 7:**
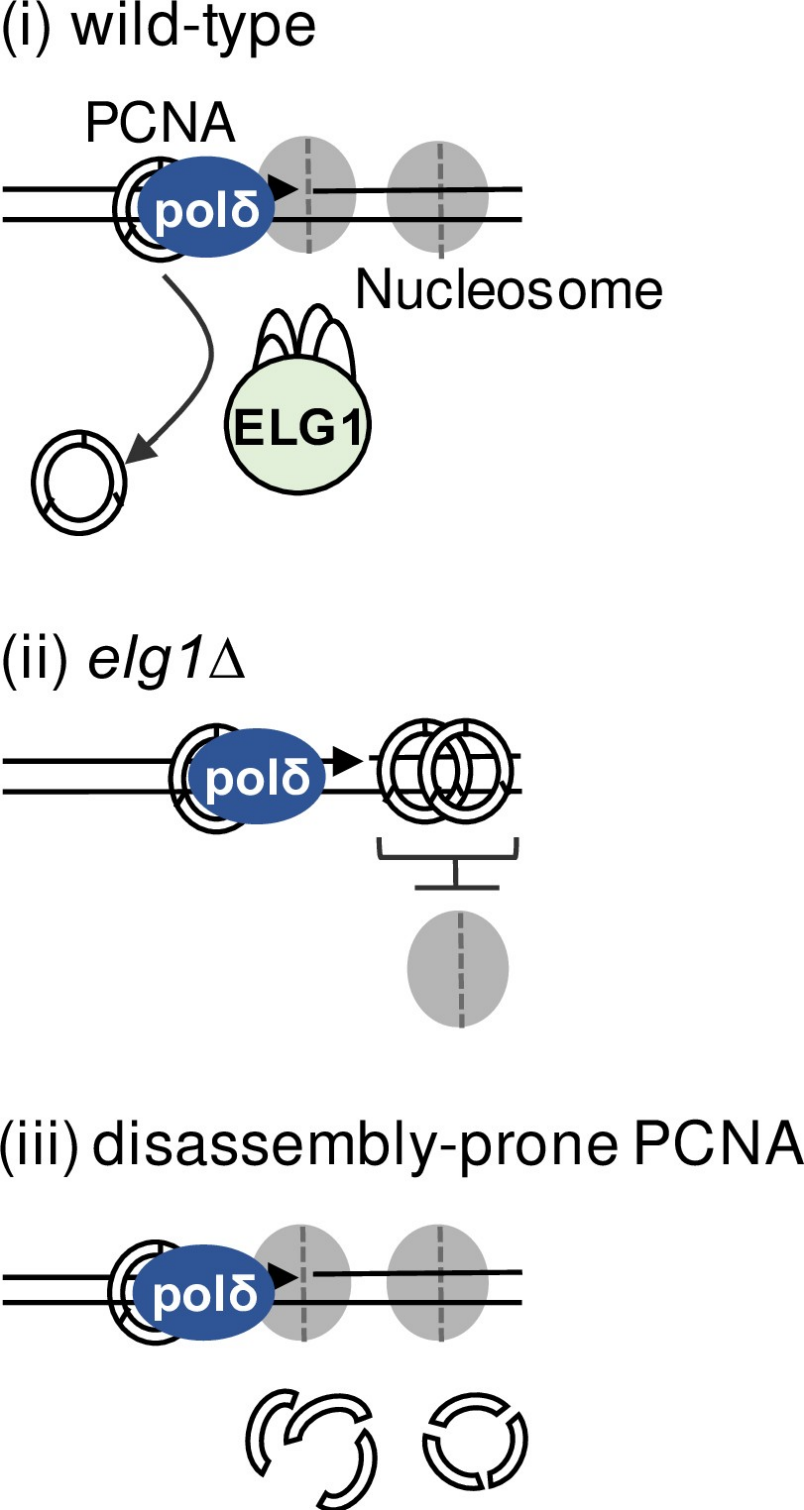
Model for the role of Elg1 in chromatin re-organization in lagging strand replication.

How might PCNA accumulation result in defective nucleosome assembly and associated Okazaki fragment lengthening? Okazaki fragment length is proposed to be regulated by nucleosome deposition on the previously synthesized section of DNA [13,38] as illustrated in Fig 7. The newly deposited nucleosome on the last piece of DNA synthesized is believed to form an obstacle to progression of polymerase δ as it carries out strand displacement synthesis, prior to completing synthesis of each Okazaki fragment. Encounter of pol δ with the nucleosome is suggested to favour pol δ disengagement and dissociation, allowing PCNA to recruit DNA ligase [39] with ligation of the completed Okazaki fragment to the nascent lagging strand determining the final Okazaki fragment length (Fig 7, Model i). We propose that in the absence of Elg1, accumulated PCNA in the wake of the replisome obstructs normal placement and spacing of nucleosome deposition, so that the nucleosomal barrier to pol δ synthesis is not present, resulting in longer Okazaki fragments being synthesized prior to their eventual completion and ligation (Fig 7, Model ii). Combining the *elg1Δ* mutation with a PCNA trimer-unstable mutant prevents the accumulation of PCNA, relieving the block to nucleosome deposition and restoring the normal mechanism of Okazaki fragment length determination (Fig 7, Model iii).

Our findings support the suggestion that nucleosome deposition is a very early event that precedes and stimulates pol δ dissociation, the polymerase in turn allowing DNA ligase recruitment by PCNA [39] and subsequent Okazaki fragment ligation, which is necessary for PCNA unloading by the Elg1-RLC. Our results are therefore consistent with the previously identified dependence of PCNA unloading on Okazaki fragment ligation [6].

A very recent study by [40] provides an interesting illustration of the consequences of disrupting PCNA removal by Elg1-RLC and nucleosome deposition. Janke et al used an assay that measures heterochromatin disruption, by testing for failure to silence expression of a Cre recombinase gene. Their finding that silencing is disrupted by an *elg1Δ* mutation (or by histone chaperone mutations) implies that normal replication-coupled chromatin assembly is needed to preserve silencing at a specific heterochromatic locus. Our study generalizes the conclusion that Elg1 activity is needed for normal chromatin inheritance, with the discovery that nucleosome deposition problems caused by failure to unload PCNA extend genome-wide.

Since delayed PCNA removal appears to be the main cause of the chromatin re-assembly defect observed in *elg1Δ*, what is the significance of Elg1 interaction with histone chaperones, in particular Rtt106 and FACT complex? Identification of these interactions raises the suggestion that Elg1 might recruit histone chaperones to assist in chromatin reassembly, with Elg1 thereby contributing to chromatin re-configuration or maturation. However, our ChIP analysis failed to identify a clear role for Elg1 in localizing Rtt106 to newly synthesized DNA. We did find that Elg1 has effects on Rtt106 chromatin association at the promoters of a number of genes, particularly genes involved in cellular transport and drug resistance. However, this effect is unlikely to be coupled to DNA replication since we observed Rtt106 association with several such sites in G1 phase samples. Slight sensitivity of an *elg1Δ* mutant to HU [8] would be consistent with a need for Elg1 in controlling the expression of genes required for drug response and export. The possibility of a non-replication-associated role for Elg1 in regulating gene expression through histone chaperone recruitment is the subject of ongoing study.

While PCNA accumulation appears to be the immediate cause of delayed nucleosome deposition in the *elg1Δ* mutant (Fig 7), our results do not exclude the possibility of a role for Rtt106-Elg1 in replication-coupled chromatin re-establishment, especially since presence of multiple, redundant histone chaperones activities in yeast complicates analysis of chromatin re-assembly. However, we could not obtain unambiguous, reproducible evidence of a role for Elg1-Rtt106 interaction following replication. One possibility is that Elg1 does contribute to coordination of chromatin re-assembly, operating through Rtt106 and/or other histone chaperones, in a pathway acting at a later stage of chromatin maturation operating after histone deposition and Okazaki fragment ligation.

The role of Elg1 appears to be conserved, since its mammalian homolog, called ATAD5, also appears to mediate PCNA unloading [41]. Mammalian cells lacking ATAD5 show PCNA accumulation on chromatin similar to that observed in yeast, and it seems likely that such PCNA retention may impact chromatin re-assembly. The major phenotype of mice lacking ATAD5 is cancer predisposition, and indeed ATAD5 mutations are also proposed to contribute to human ovarian cancers [42,43]. Defects in genomic function caused by derailed chromatin re-assembly following replication might therefore contribute significantly to human cancer development or progression.

## Materials and methods

### Yeast strains

All yeast strains used in this study are listed in S1 Table. Gene disruptions and epitope tags were introduced by standard PCR based methods [44,45].

### Detection of Okazaki fragments

Okazaki fragment purification and detection was performed as described previously in [13].

### Analysis of nascent chromatin structure by micrococcal nuclease digestion

Yeast cells were grown to OD_600_ of 0.2 at 30°C in 60ml YPD media and then alpha factor was added to arrest cells in G1 phase. 400μg/ml BrdU was added to the culture and incubated for 30 minutes for cells to take up BrdU. Cells were then released into S phase by resuspending in fresh YPD containing 400μg/ml BrdU. Then 20 ml samples were collected at desired time points into formaldehyde (1% final concentration) and incubated with rotation for 15 minutes at room temperature. 125mM glycine was then added to neutralise formaldehyde. Cells were washed twice in 10 ml of ice cold 1X PBS, then with 2 ml of spheroplasting buffer (1M sorbitol, 1mM beta-mercaptoethanol) before resuspending in 1ml of spheroplasting buffer with 300μg/ml 100-T Zymolase then incubated at 30°C for 20 minutes. Spheroplasts were washed in 1ml of spheroplasting buffer and resuspended in 600μl of Digestion buffer (1M sorbitol, 50mM Nacl, 10mM Tris-HCl pH7.4, 5mM MgCl_2_, 5mM CaCl_2_, 0.075% Nonidet P-40, 1mM beta-mercaptoethanol, 0.5mM spermidine). 200μl aliquots were subjected to micrococcal nuclease (NEB, M0247S) digestion (200 or 600 gel units) for 5 minutes at 37°C. Digestions were stopped by adding 1/10 volume of stop solution (250mM EDTA, 5% SDS). 5μl of 20 mg/ml Proteinase K was added and incubated overnight at 55°C. Following phenol-chloroform extraction, DNA was precipitated using 1/10 volume of 3M sodium acetate and 2 volumes of 100% ethanol. The air-dried DNA pellet was resuspended in 20μl of TE buffer with RNase A (1mg/ml) and incubated for 2 hours at 37°C. DNA samples were electrophoresed on a 1.4% agarose gel, which was incubated in denaturing buffer (0.5M NaoH, 1M NaCl) twice for 15 minutes followed by incubation in neutralization buffer (0.5M Tris-Hcl, 3M Nacl) for 30 minutes. The DNA was then transferred to Amersham Hybond N^+^ membrane by Southern blotting. DNA was cross-linked to the membrane with UV light (1200J). The membrane was then incubated in 5% milk in TBS-tween for 60 minutes and probed with anti-BrdU antibody (ab12219, abcam).

### Protein affinity techniques

Whole cell extract preparation, western blotting and co-immunoprecipitation experiments were performed as described previously [5,6]. Antibodies used were: anti-BrdU (ab12219, abcam), anti-FLAG (F1804, Sigma), anti-HA (HA.11 clone 16B12, Covance), anti-PCNA (ab70472, abcam).

### SILAC-mass spectrometry

SILAC Quantitative proteomic analysis was performed as described previously [46].

### ChIP-seq analysis

Yeast strains were grown to an OD_600_ of 0.25 in YPD. Alpha factor was added to arrest cells in G1 and released into YPD containing 0.2M hydroxyurea at 23°C for 60 minutes. Formaldehyde (1% final concentration) was added and incubated with rotation first at room temperature for 20 minutes and then at 4°C overnight. Cells were washed 3 times with ice-cold 1X Phosphate buffered saline. Cells were pelleted and frozen at -80°C. Rtt106 ChIP using anti-HA (HA.11 clone 16B12, Covance) and data analysis were performed as described previously [6].

ChIP-Seq data are uploaded to Array Express under accession number: E-MTAB-6985

### MNase-seq analysis

#### Chromatin digestion and EdU pulldown

Yeast cells were grown to an OD_600_ of 0.66 at 30°C, then incubated with alpha factor for 2.5 hours at 30°C. Cells were then released into YPD containing 50μM EdU at room temperature. Samples were collected at indicated time points into formaldehyde (1% final concentration) and incubated for 10 minutes, followed by quenching with 125 mM glycine for 5 minutes. Cells were chilled on ice, washed 3X with cold Tris-buffered saline (20mM Tris pH 7.5, 120 mM NaCl), pelleted and frozen at -80°C. Frozen cell pellets were resuspended in ice cold Spheroplast Digestion Buffer (1M Sorbitol, 50mM NaCl, 10mM Tris-HCl pH 7.5, 5mM MgCl2, 1mM CaCl2, and 0.075% v/v Nonidet P-40 Substitute supplemented with 1mM 2-mercaptoethanol, and 0.5mM of each of the following: Spermidine, Pepstatin, Aprotinin, benzamidine, E64, and ABSF) then lysed in a Mini-Bead Beater 8 at 4°C. MNase was titrated to give 80% mononucleosomal DNA bands, typically 30 units for 15 minutes at 37°C. Samples were incubated overnight at 65°C with proteinase K and the supernatant was treated with RNase A for 1h at 37°C, followed by phenol-chloroform extraction and isopropanol precipitation. Excised mononucleosome bands from an agarose size-selection gel were purified using Freeze-n-Squeeze columns followed by phenol-chloroform extraction and cold ethanol precipitation.

A Click-iT NascentRNACapture Kit (Invitrogen, C10365) was used to biotinylate EdU-labelled nascent DNA, which was pulled down with Streptavidin MyOne Dynabeads (Invitrogen, 65601) according to Invitrogen protocols.

#### High throughput sequencing library construction & Data analysis

DNA was blunt-ended, an A-overhang was added, and Illumina adapters were ligated on, with Agencourt Ampure XP bead washes after each step. PCR amplification was performed with 18 or fewer cycles using Illumina barcoded primers and primer PE 1.0, followed by a final Ampure bead purification. Adapter Sequences used: Top PE Adapter (ACACTCTTTCCCTACACGACGCTCTTCCGATC*T) Bottom PE Adapter (P-GATCGGAAGAGCGGTTCAGCAGGAATGCCGAG) PE 1.0 (AATGATACGGCGACCACCGAGATCTACACTCTTTCCCTACACGACGCTCTTCCGATCT).

Reads were mapped to the *S*. *cerevisiae* genome using Bowtie2. Replication origin [47] and TSS data was generated and graphed using custom Python scripts, with strand orientation accounted for in the analysis. Data were normalized by dividing by mean read depth per base pair and plots were smoothed with a 50bp sliding window.

MNase-Seq data are uploaded to Array Express under accession number: E-MTAB-6985

## Supporting information

S1 Fig Biological repeat of Fig 1C. Okazaki Fragment length assay showing Okazaki fragments are extended in *elg1Δ*, similar to *cac2Δ* and unlike *ctf18Δ*. Dotted lines show Okazaki fragments corresponding to mono- and di-nucleosome sizes. Trace of signal intensity for each lane is shown.
(TIF)

S2 Fig Micrococcal nuclease digestion of nascent chromatin reveals defective nucleosome assembly in *elg1Δ* (**A**) & **(B**) and *cac1Δ* (**C**) compared to WT. Signal traces represent 45 min nascent DNA sample lanes (highest concentration of MNase lane) revealing increased mononucleosomal DNA in the mutants when compared to WT. MNase digestion experiments were performed as described in Fig 2. Panel A & B shows biological repeats of Fig 2C.
(TIF)

S3 Fig Flow cytometry profiles show no difference in S phase progression in *WT* and *elg1Δ*. Cells were arrested in G1 using alpha factor and released into S phase at 30°C and samples were collected at indicated time-points for flow-cytometry analysis.
(TIF)

S4 Fig Genome-wide MNase-seq analysis shows defective nucleosome organization in *elg1Δ (***A**) and *cac1Δ* (**B**). Nascent DNA nucleosomal reads (as in Fig 3) aligned to Transcription Start Sites (TSS). G1 samples show total DNA, and 27.5–60 min samples nascent DNA recovered by EdU pulldown. Plots in panel A show the mean of two biological repeats, whereas plots in panel B are from one experiment.
(TIF)

S5 Fig Biological replicates (**A** & **B**) showing genome-wide MNase-seq analysis, revealing defective nucleosome organization in *elg1Δ*. Nucleosomal reads on nascent DNA aligned to replication origins (ARS). G1 samples show total DNA, whereas 27.5–60 min samples show nascent DNA recovered by EdU pulldown. Fig 3B shows the mean of these two biological repeats.
(TIF)

S6 Fig Both N-terminal and C-terminal domains of Elg1 are important for interaction with Rtt106. **A**. Immunoprecipitation experiment to map domains of Elg1 interacting with Rtt106. WT Elg1 and Elg1 fragments were expressed from the endogenous locus and promoter. Elg1-3FLAG immunoprecipitation was performed as described previously [11]. PCNA interaction data as shown in [11]. **B**. Schematic structure of Elg1 and truncation mutants, with strength of Rtt106 interaction indicated.
(TIF)

S7 Fig Co-immunoprecipitation under different salt concentrations (potassium acetate as indicated) shows interaction of Elg1 with Rtt106 is not mediated by PCNA. Asterisk denotes degradation product.
(TIF)

S8 Fig PCNA on chromatin is slightly increased in *rtt106Δ* compared to WT, but not to the extent of *elg1Δ*. (A). Whole cell extract and chromatin fractions from indicated strains were prepared and analysed by western blotting. Plots showing quantification of the relative amounts of unmodified PCNA in Whole Cell Extract (WCE) and Chromatin (Ch) (**B**) and K164-SUMO PCNA (**C**) in the mutant strains compared to WT. K164-SUMO PCNA is a marker of chromatin association. Whole cell extract and chromatin-enriched fraction prepared as described previously [6].
(TIF)

S9 Fig ChIP-Seq experiment showing Rtt106-6HA recruitment at promoter region of *HTA1-HTB1* (**A**) and origins of replication (**B**).
(TIF)

S10 Fig Biological replicate of the experiment shown in Fig 6. Disassembly-prone mutant of PCNA (*pol30-R14E* or *pol30-D150E*) rescue the Okazaki fragment length extension observed in *elg1Δ*. Dotted lines show Okazaki fragments corresponding to mono- and di-nucleosome sizes. Trace of signal intensity for each lane is shown.
(TIF)

S1 Table Yeast strains used in this study. (XLSX)

S1 Dataset SILAC-based quantitative mass spectrometry analysis for identification of Elg1 interaction partners. (XLSX)
