# Supplementary figures and images for: Identification of Elg1 interaction partners and effects on post-replication chromatin re-formation
Source: PLoS Genet. 2018 Nov 12;14(11):e1007783. doi: 10.1371/journal.pgen.1007783 (PMC6258251; doi:10.1371/journal.pgen.1007783)

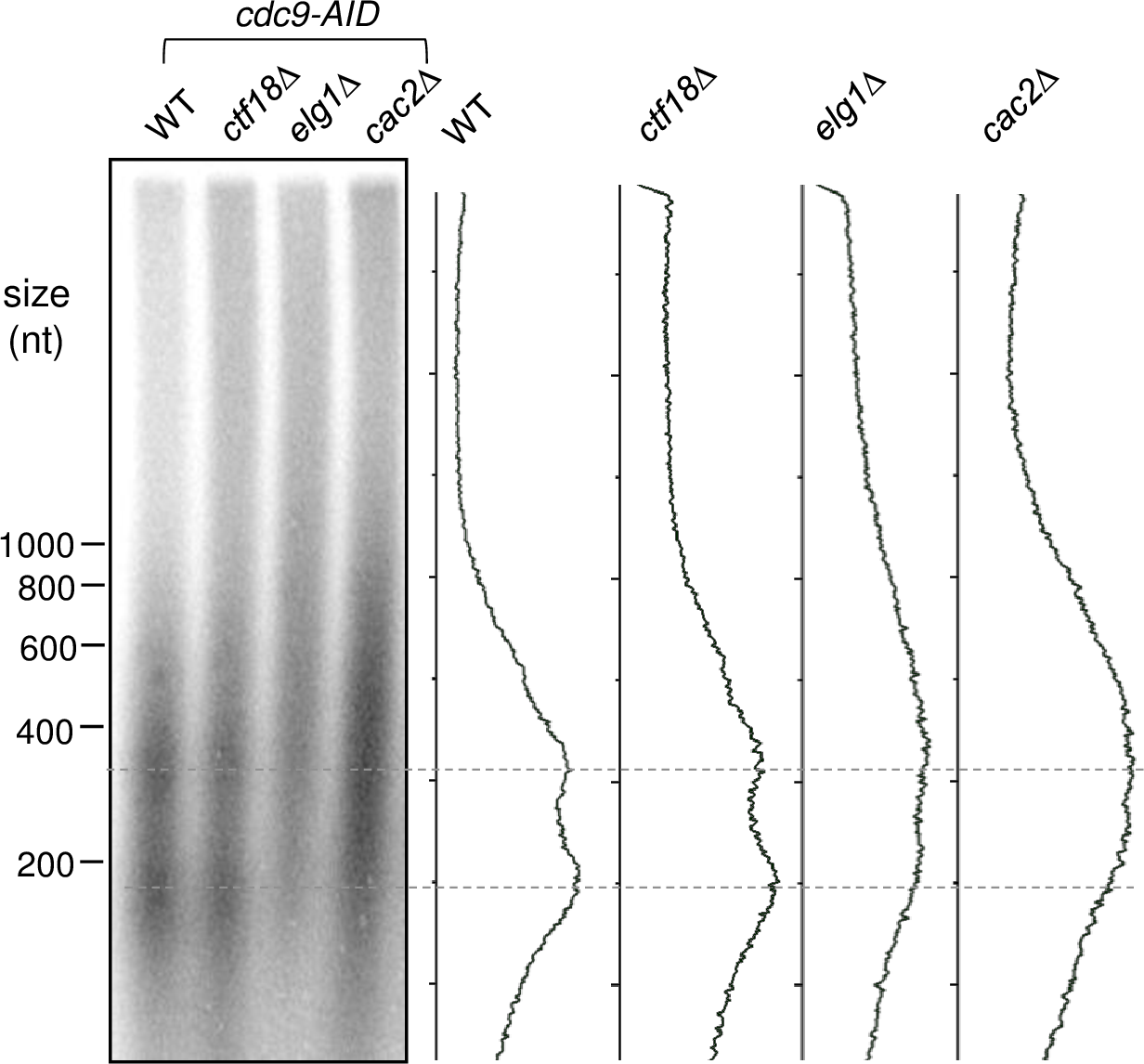

Supplement: S1 Fig — Okazaki Fragment length assay showing Okazaki fragments are extended in elg1Δ, similar to cac2Δ and unlike ctf18Δ. Dotted lines show Okazaki fragments corresponding to mono- and di-nucleosome sizes. Trace of signal intensity for each lane is shown. (TIF) [file pgen.1007783.s001.tif]

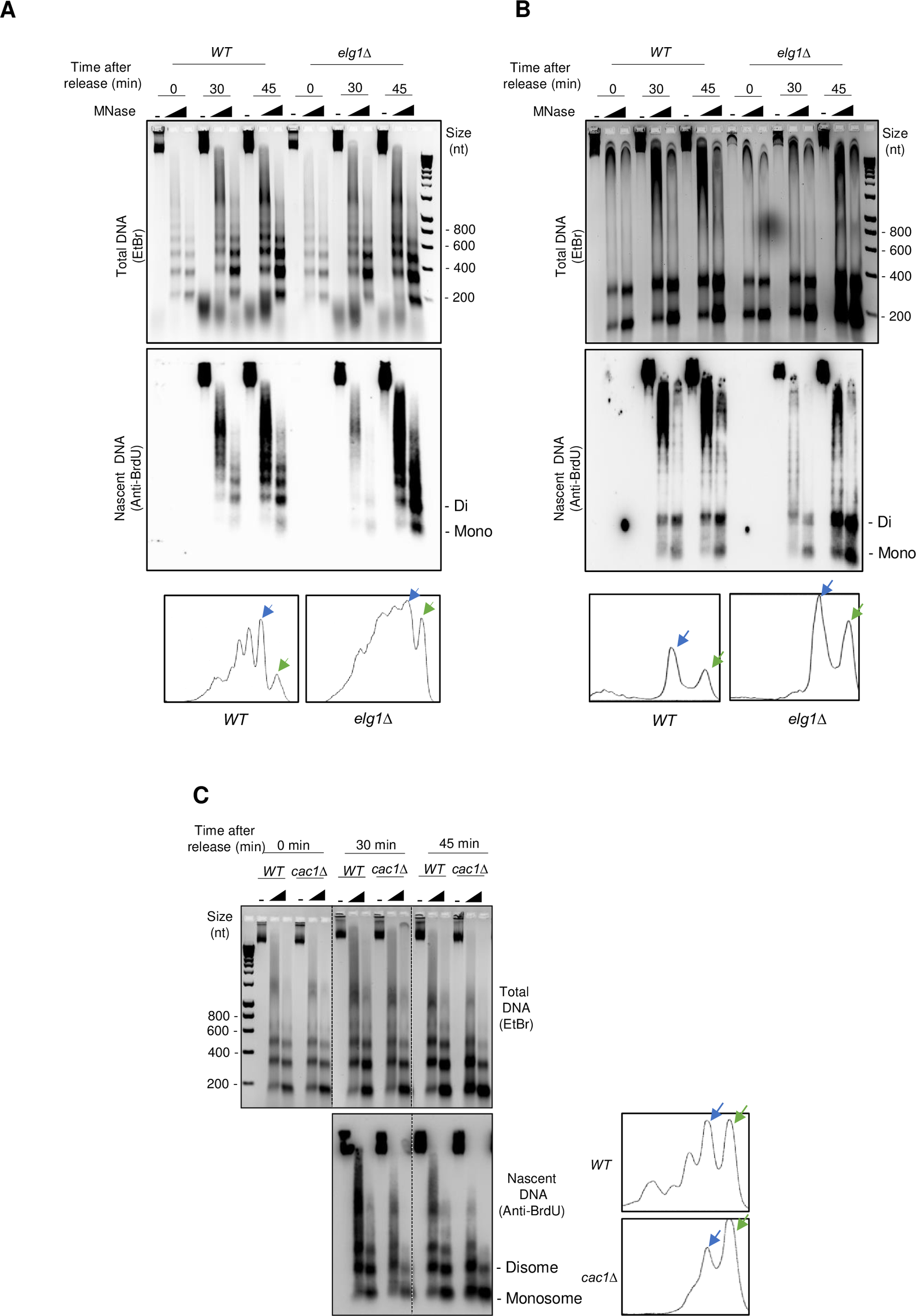

Supplement: S2 Fig — Micrococcal nuclease digestion of nascent chromatin reveals defective nucleosome assembly in elg1Δ (A) & (B) and cac1Δ (C) compared to WT. Signal traces represent 45 min nascent DNA sample lanes (highest concentration of MNase lane) revealing increased mononucleosomal DNA in the mutants when compared to WT. MNase digestion experiments were performed as described in Fig 2. Panel A & B shows biological repeats of Fig 2C. (TIF) [file pgen.1007783.s002.tif]

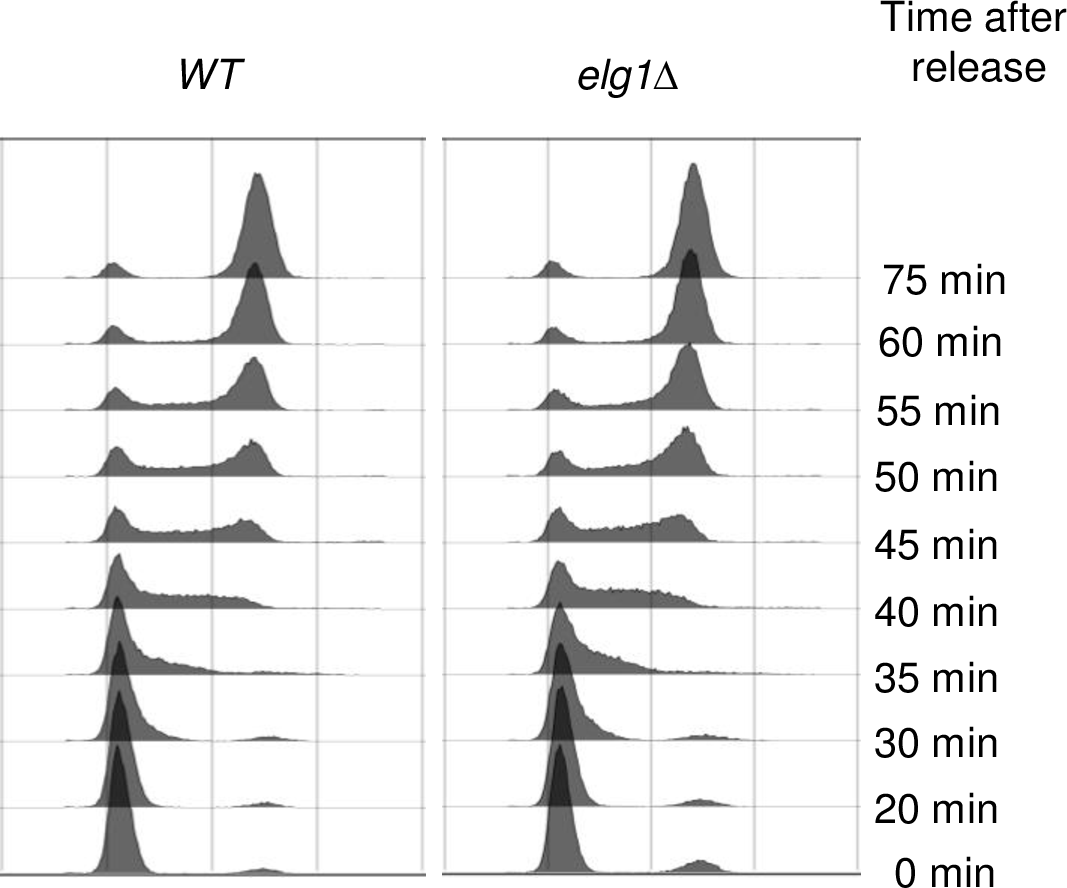

Supplement: S3 Fig — Cells were arrested in G1 using alpha factor and released into S phase at 30°C and samples were collected at indicated time-points for flow-cytometry analysis. (TIF) [file pgen.1007783.s003.tif]

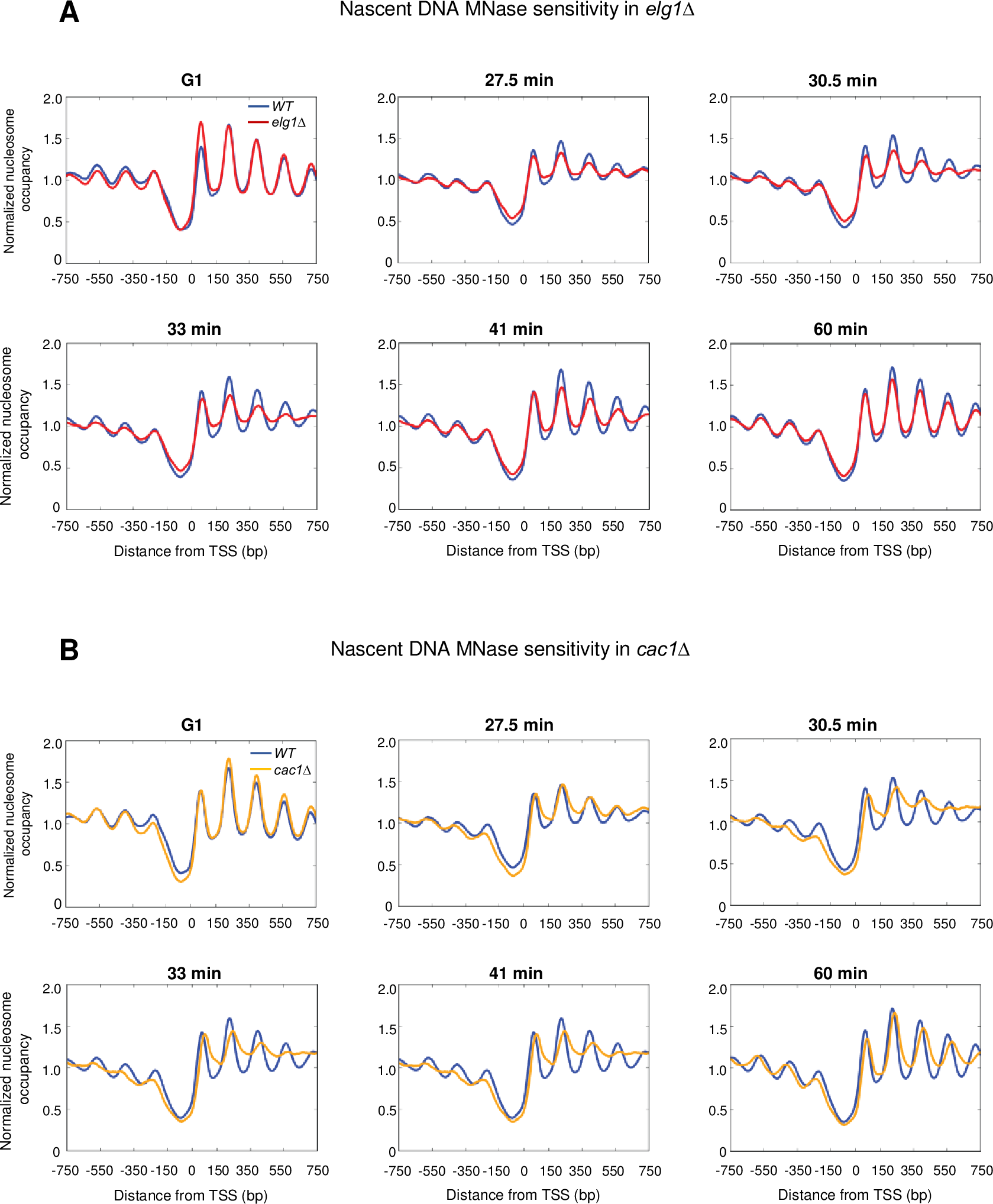

Supplement: S4 Fig — Genome-wide MNase-seq analysis shows defective nucleosome organization in elg1Δ (A) and cac1Δ (B). Nascent DNA nucleosomal reads (as in Fig 3) aligned to Transcription Start Sites (TSS). G1 samples show total DNA, and 27.5–60 min samples nascent DNA recovered by EdU pulldown. Plots in panel A show the mean of two biological repeats, whereas plots in panel B are from one experiment. (TIF) [file pgen.1007783.s004.tif]

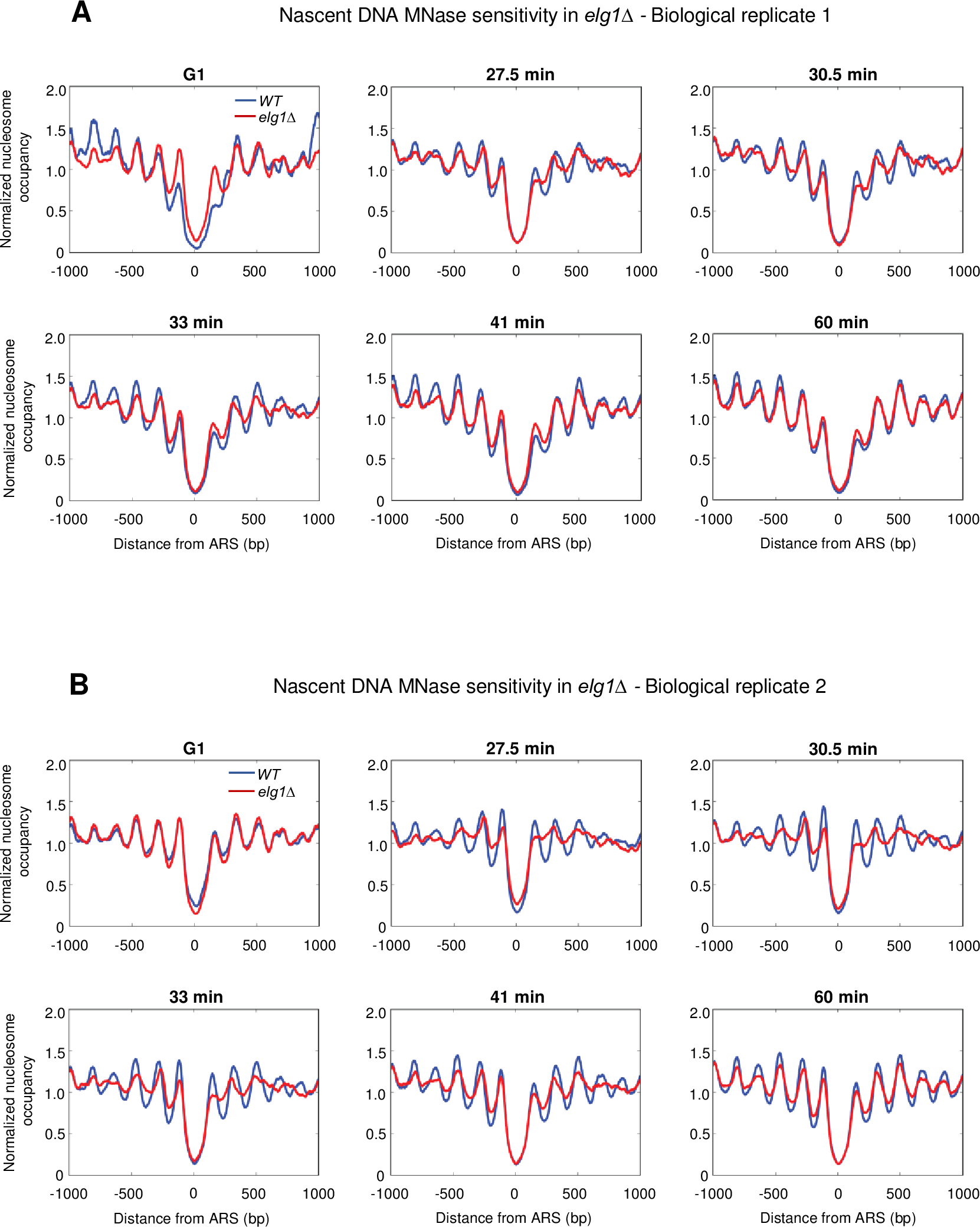

Supplement: S5 Fig — Biological replicates (A & B) showing genome-wide MNase-seq analysis, revealing defective nucleosome organization in elg1Δ. Nucleosomal reads on nascent DNA aligned to replication origins (ARS). G1 samples show total DNA, whereas 27.5–60 min samples show nascent DNA recovered by EdU pulldown. Fig 3B shows the mean of these two biological repeats. (TIF) [file pgen.1007783.s005.tif]

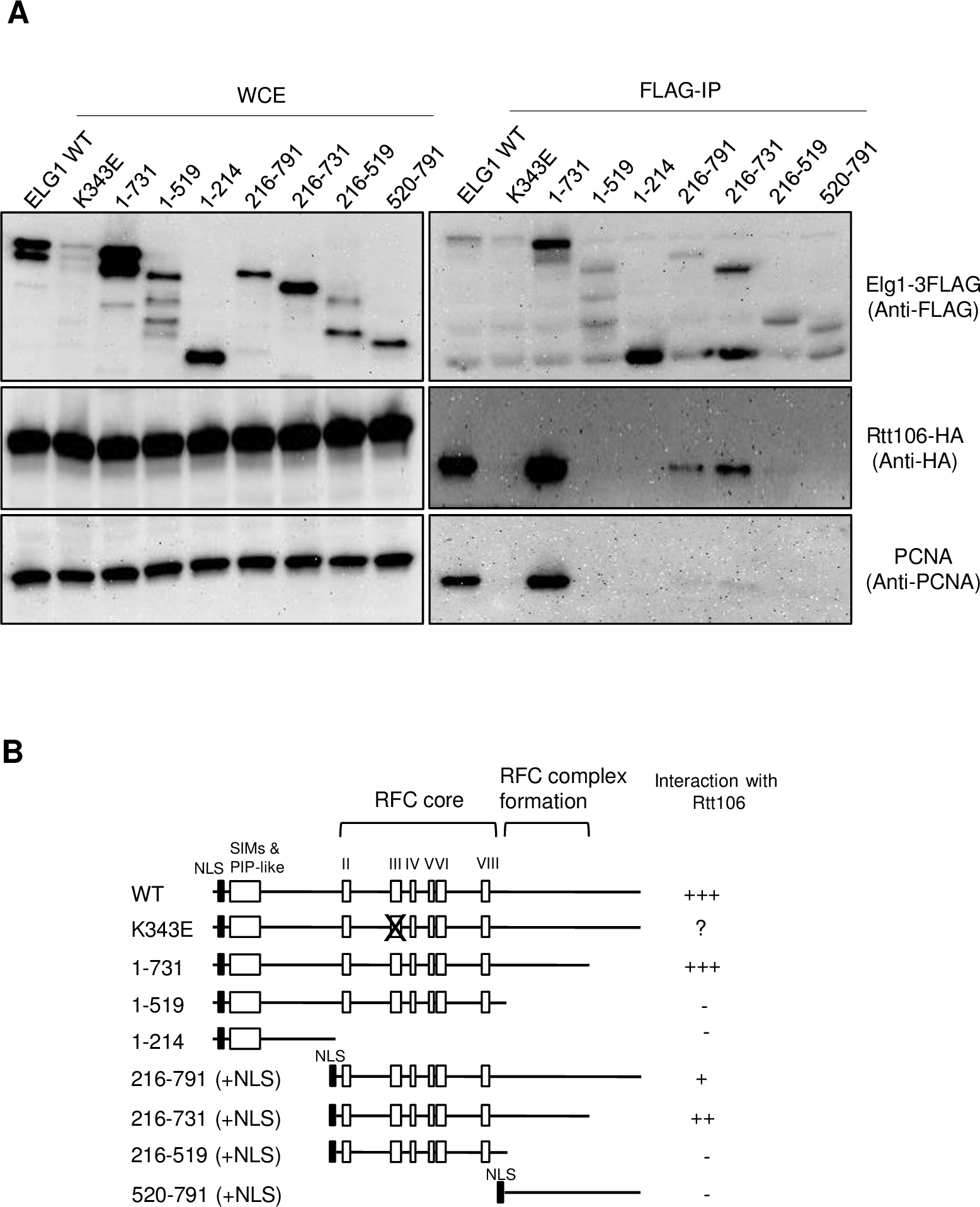

Supplement: S6 Fig — A. Immunoprecipitation experiment to map domains of Elg1 interacting with Rtt106. WT Elg1 and Elg1 fragments were expressed from the endogenous locus and promoter. Elg1-3FLAG immunoprecipitation was performed as described previously [11]. PCNA interaction data as shown in [11]. B. Schematic structure of Elg1 and truncation mutants, with strength of Rtt106 interaction indicated. (TIF) [file pgen.1007783.s006.tif]

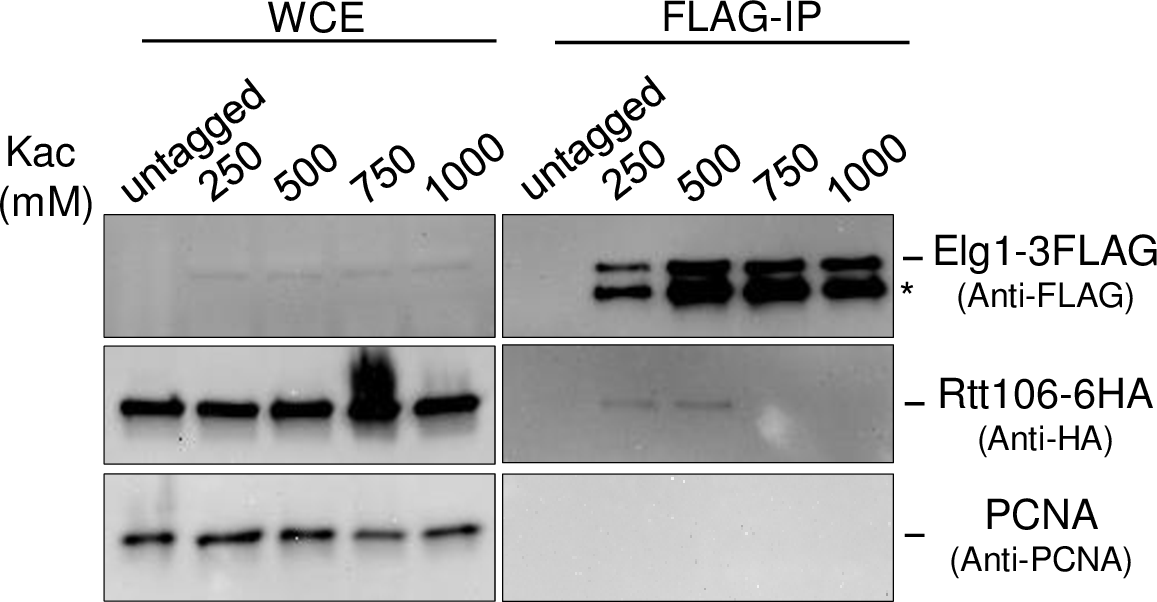

Supplement: S7 Fig — Asterisk denotes degradation product. (TIF) [file pgen.1007783.s007.tif]

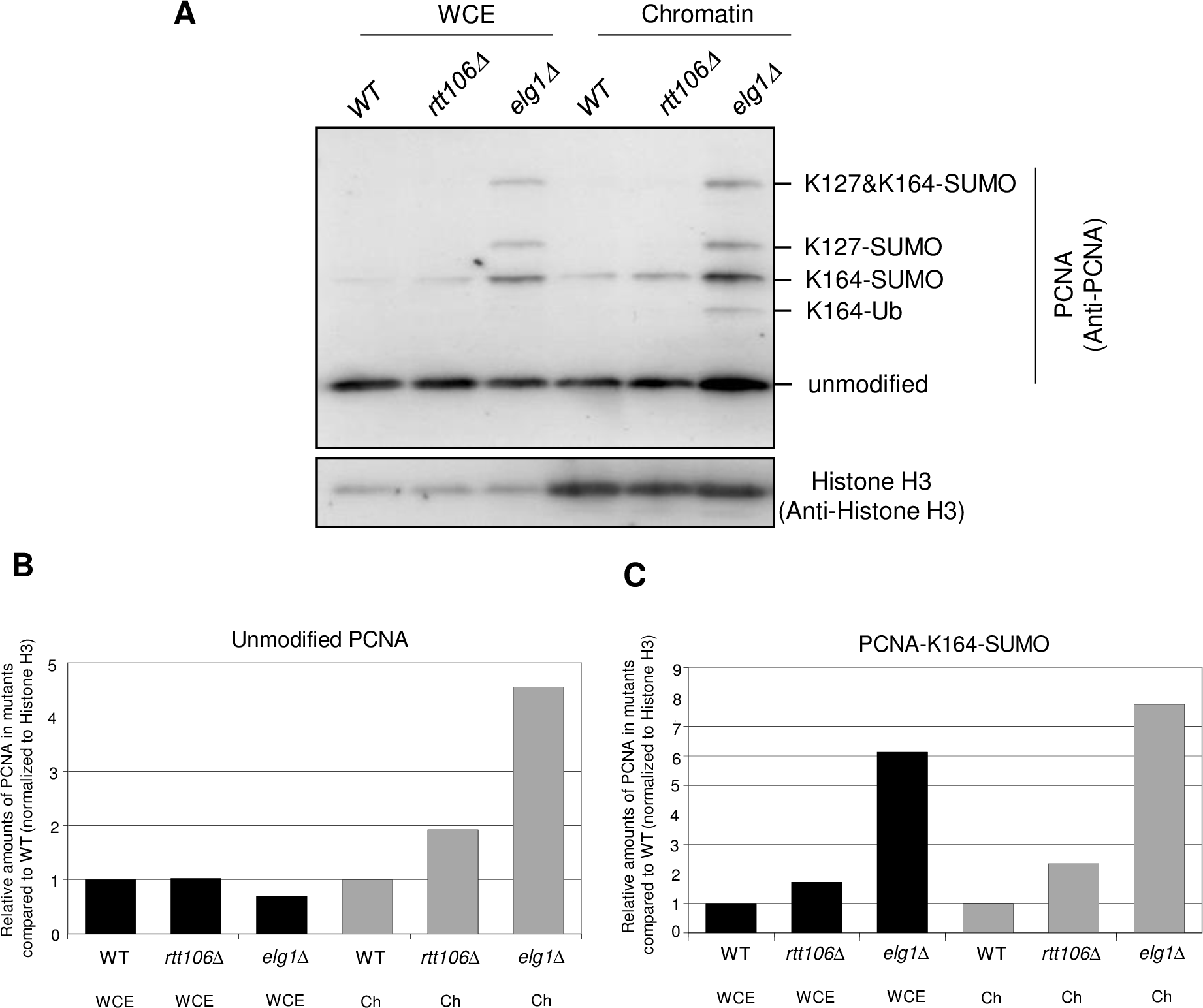

Supplement: S8 Fig — (A). Whole cell extract and chromatin fractions from indicated strains were prepared and analysed by western blotting. Plots showing quantification of the relative amounts of unmodified PCNA in Whole Cell Extract (WCE) and Chromatin (Ch) (B) and K164-SUMO PCNA (C) in the mutant strains compared to WT. K164-SUMO PCNA is a marker of chromatin association. Whole cell extract and chromatin-enriched fraction prepared as described previously [6]. (TIF) [file pgen.1007783.s008.tif]

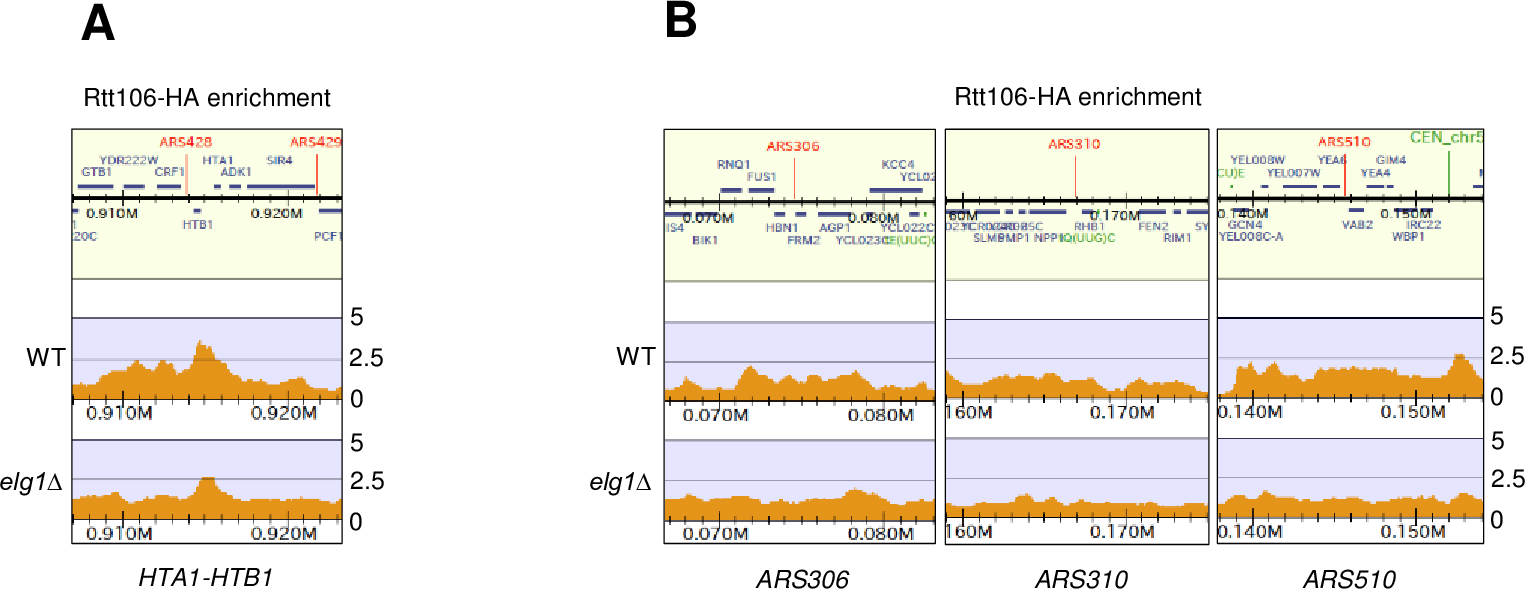

Supplement: S9 Fig — ChIP-Seq experiment showing Rtt106-6HA recruitment at promoter region of HTA1-HTB1 (A) and origins of replication (B). (TIF) [file pgen.1007783.s009.tif]

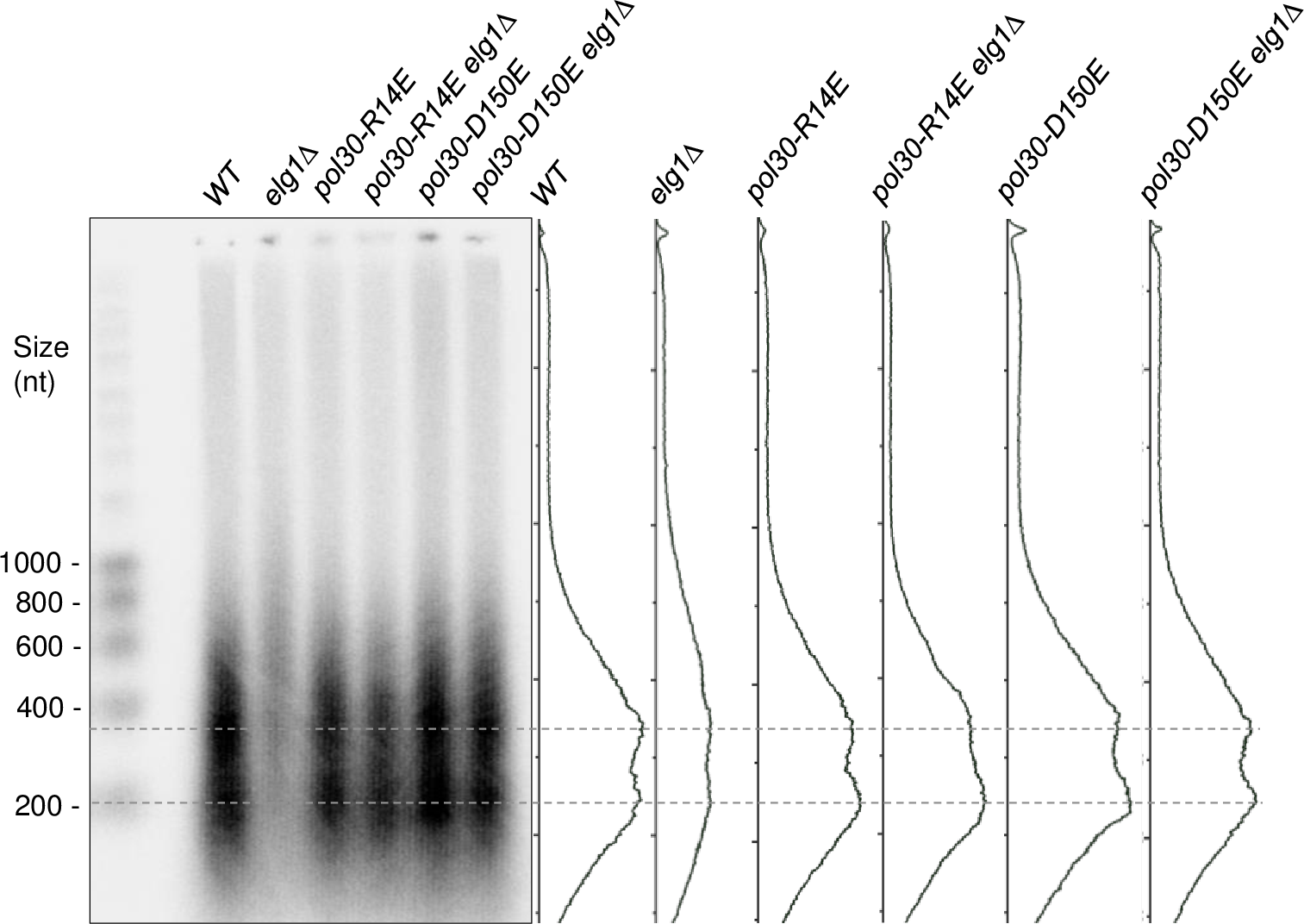

Supplement: S10 Fig — Disassembly-prone mutant of PCNA (pol30-R14E or pol30-D150E) rescue the Okazaki fragment length extension observed in elg1Δ. Dotted lines show Okazaki fragments corresponding to mono- and di-nucleosome sizes. Trace of signal intensity for each lane is shown. (TIF) [file pgen.1007783.s010.tif]
